# Serological biomarker models composed of luteinizing hormone, kisspeptin, vitamin D and estradiol, and their clinical test value in girls

**DOI:** 10.1016/j.jped.2025.101477

**Published:** 2025-11-22

**Authors:** Mingyu Shao, Zhiyi Jia, Tongtong Liu, Xiaopeng Zhang

**Affiliations:** a Zibo Central Hospital, Department of Child Health Care, Shandong, China; b Zibo Central Hospital, Department of Pediatrics, Shandong, China

**Keywords:** Central precocious puberty, Luteinizing hormone, Kisspeptin, Vitamin D, Estradiol

## Abstract

**Objective:**

Central precocious puberty (CPP) may lead to premature pubertal onset. While the GnRH stimulation test remains the gold standard, its invasive nature and prolonged procedure limit clinical utility, particularly in pediatric populations. Emerging biomarkers, including luteinizing hormone, estradiol, kisspeptin, and vitamin D, have shown promise in distinguishing CPP from normal puberty. This study systematically evaluated the diagnostic utility of these biomarkers both individually and in novel combinatorial models, with the aim of establishing a non-invasive and more accessible diagnostic alternative for CPP.

**Methods:**

This retrospective study included 129 girls with CPP and 116 age-matched controls. Clinical characteristics, including height, weight, body mass index, and bone age, were recorded. Serum levels of luteinizing hormone, estradiol, kisspeptin, vitamin D, progesterone, and prolactin were measured. The diagnostic performance of individual biomarkers and three combined biomarker models was assessed using receiver operating characteristic curve analysis.

**Results:**

Model 1 included luteinizing hormone and kisspeptin, Model 2 incorporated vitamin D, and Model 3 added estradiol. CPP patients exhibited significantly higher levels of luteinizing hormone (2.51 vs. 0.23 mIU/mL), kisspeptin (1.59 vs. 0.96 μg/L), and estradiol (25.86 vs. 13.41 pg/mL) and lower vitamin D levels (20.13 vs. 25.90 ng/mL) compared to controls (all *p* < 0.001). Model 3 demonstrated the highest diagnostic accuracy with an AUC of 0.939 (95 % CI: 0.910–0.968), sensitivity of 89.06 %, and specificity of 87.93 %, outperforming individual biomarkers and other models.

**Conclusion:**

This study highlights the potential of combining luteinizing hormone, kisspeptin, vitamin D, and estradiol into a single diagnostic model for CPP.

## Introduction

Central precocious puberty (CPP) is a condition marked by the premature activation of the hypothalamic-pituitary-gonadal axis, leading to the early onset of secondary sexual characteristics before the age of 8 years in girls and 9 years in boys [1]. CPP is significantly more common in girls and has far-reaching implications for physical, psychosocial, and emotional development, including advanced bone maturation, reduced adult height, and emotional challenges [1,2]. Diagnosing CPP accurately is critical for initiating timely interventions, such as gonadotropin-releasing hormone (GnRH) analog therapy, which can mitigate long-term consequences of early puberty, and to date, the GnRH stimulation test remains the gold standard method for diagnosing CPP [3]. However, distinguishing CPP from other variants of early puberty, such as isolated premature thelarche or non-progressive puberty, remains a diagnostic challenge [4].

Traditionally, CPP diagnosis has relied on clinical features, radiographic evidence of advanced bone age, and hormonal evaluations, including basal and stimulated gonadotropin levels. However, the GnRH stimulation test, although sensitive, is resource-intensive, time-consuming, and inconvenient for pediatric patients [5]. Additionally, gonadotropin levels often overlap between CPP and other conditions, complicating the diagnosis [6]. These limitations highlight the unmet need for non-invasive and reliable biomarkers that can improve diagnostic accuracy. In this context, devising a new diagnostic model with higher specificity would be preferred to reduce unnecessary GnRH testing in these benign cases.

Recent studies have explored the utility of serum biomarkers such as luteinizing hormone, estradiol, and progesterone, which are central to the activation of the hypothalamic-pituitary-gonadal axis [7,8]. Emerging biomarkers such as kisspeptin, a neuropeptide regulating gonadotropin-releasing hormone secretion, have shown promise in distinguishing CPP cases [9]. Additionally, vitamin D, which is inversely associated with puberty progression, offers potential as a diagnostic biomarker [10,11]. However, the diagnostic utility of these biomarkers remains limited by variability in sample collection times, pubertal stages, and individual differences [12]. Furthermore, few studies have evaluated combinations of biomarkers to enhance diagnostic accuracy.

By leveraging receiver operating characteristic (ROC) curve analysis, researchers can systematically evaluate the diagnostic performance of individual biomarkers and biomarker models in terms of sensitivity, specificity, and area under the curve (AUC) [13]. Although individual biomarkers provide important insights, their diagnostic utility is often limited when used in isolation. Integrating biomarkers into combined models has shown potential for improving diagnostic performance, but this approach remains underexplored for CPP diagnosis.

This study aims to address these limitations by evaluating the clinical and serological profiles of girls with CPP and assessing the diagnostic value of key biomarkers, including luteinizing hormone, estradiol, vitamin D, and kisspeptin. By constructing and validating three serological biomarker models, this study seeks to identify a non-invasive, efficient, and accurate diagnostic tool for distinguishing CPP from normal puberty. The findings are expected to contribute to the growing body of evidence supporting biomarker-based diagnostics for managing CPP [14].

## Methods

### Study design and participants

This study, being retrospective in nature, was conducted using data from 129 girls diagnosed with central precocious puberty (CPP) who were treated at Zibo Central Hospital. The control group included 116 age-matched girls who underwent routine health examinations during the same period. The diagnostic criteria for ICPP were based on the guidelines referenced from the Chinese Medical Association: (1) Onset of secondary sexual characteristics in girls before age 8 years or menarche occurring under age 10 years; (2) Accelerated linear growth, bone age (BA) exceeding chronological age by 1 year or more; (3) Enlargement of the gonads: pelvic ultrasound shows increased uterine and ovarian volume in girls, with multiple ovarian follicles > 4 mm in diameter; (4) Activation of the HPGA: The serum levels of gonadotropins and sex hormones reached pubertal levels. Exclusion criteria comprised: (1) Secondary central precocious puberty with a clearly defined organic cause; (2)Participants taking known medications affecting the HPGA or having used steroid medications prior to the study; (3) Peripheral precocious puberty; (4) Known endocrine diseases or chromosomal abnormalities were excluded from the study; (5) Severe organic diseases and incomplete medical records; (6) Individuals with corneal inflammation, blepharitis, conjunctival stones, entropion, and abnormal blinking due to local eye diseases and ocular irritation factors were excluded; (7) Those with abnormal blinking resulting from refractive errors, drugs, psychological, and systemic illnesses were also excluded; (8) Individuals with a history of eye trauma, surgery, or corneal contact lens wear were not included. The participant selection process is outlined in the study flowchart. The study was approved by Zibo Central Hospital, the approval number was 2010-SMY. The study was performed in strict accordance with the Declaration of Helsinki, Ethical Principles for Medical Research Involving Human Subjects. Written informed consent was waived due to the nature of the retrospective analysis.

### Clinical and biomarker measurements

Baseline clinical characteristics, including height, weight, body mass index (BMI), and bone age, were recorded for all participants. Blood samples were collected, and serum levels of basal luteinizing hormone, progesterone, estradiol, prolactin, vitamin D, and kisspeptin were measured using standardized assays. The concentrations of these biomarkers were compared between the CPP and control groups.

### Biomarker model development and evaluation

ROC curve analysis was conducted to evaluate the diagnostic performance of individual biomarkers, including luteinizing hormone, estradiol, vitamin D, and kisspeptin, in distinguishing CPP cases from controls. Serological biomarker models were developed to assess combined diagnostic utility. Model 1 included luteinizing hormone and kisspeptin, Model 2 incorporated luteinizing hormone, kisspeptin, and vitamin D, and Model 3 included luteinizing hormone, kisspeptin, vitamin D, and estradiol. ROC curves were generated for each model, and diagnostic performance was assessed by calculating the area under the curve (AUC), 95 % confidence intervals (CI), sensitivity, specificity, and cut-off values. These parameters were compared to determine the diagnostic utility of each model. To verify the scientific validity of the study design, the selection of this specific combination was based on step-wise logistic regression.

### Statistical analysis

Continuous variables were expressed as medians with interquartile ranges, and categorical variables as frequencies and percentages. Between-group comparisons were performed using the Mann-Whitney U test for continuous data and the chi-squared test for categorical data. ROC curve analysis was used to evaluate the diagnostic performance of individual biomarkers and combined models, and AUC values were compared using statistical software. Statistical significance was set at *p* < 0.05. All analyses were performed using SPSS.

## Results

### Baseline characteristics indicate accelerated growth and maturation in CPP

**Figure 1:**
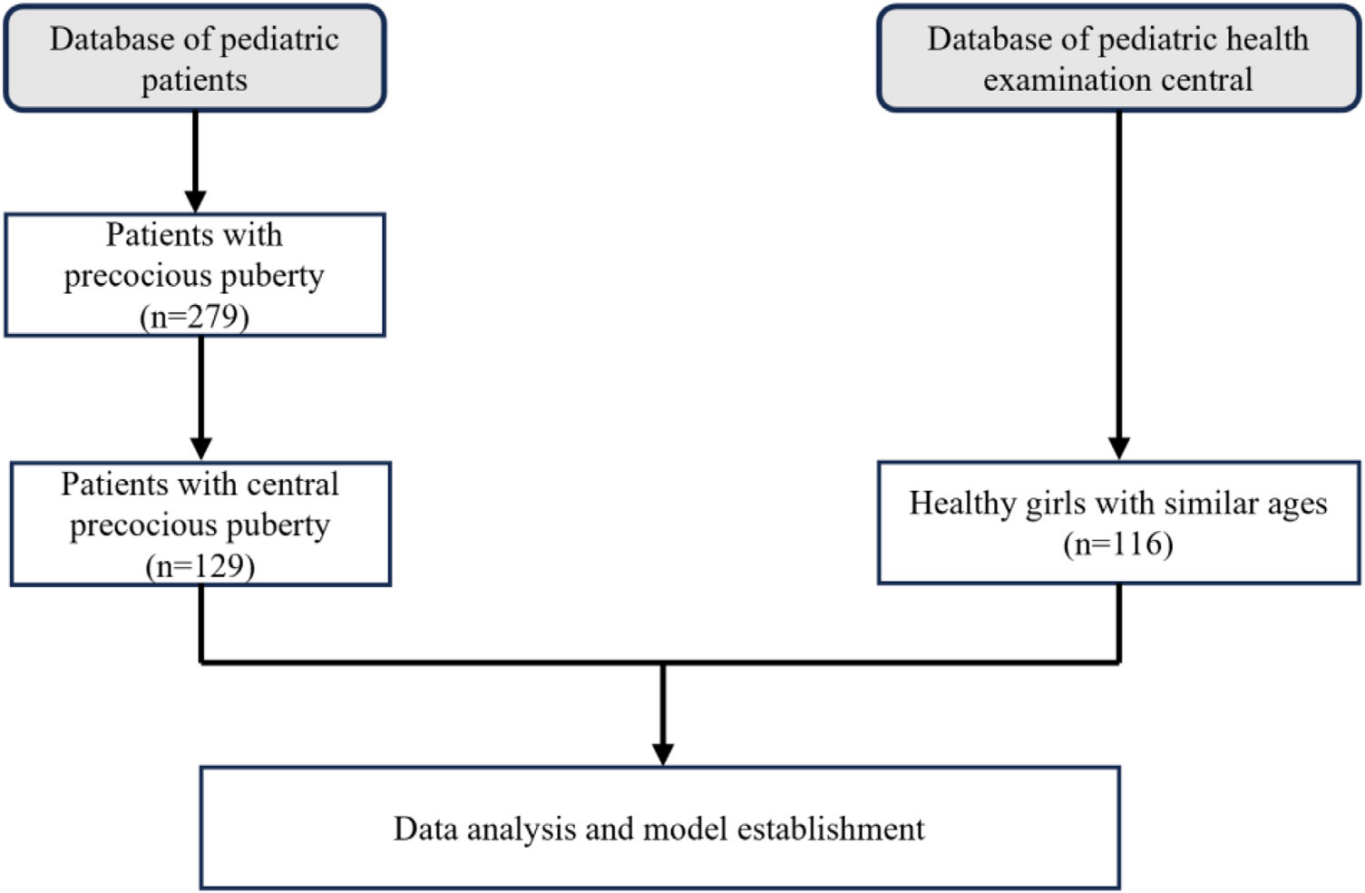
Flow chart of the study participants.

A total of 245 girls were included in the study, with 116 in the control group and 129 in the CPP group. The participant selection and group allocation are detailed in Figure 1. The baseline clinical characteristics, shown in Table 1, revealed no significant difference in chronological age between the two groups (*p* = 0.991). However, girls in the CPP group exhibited significantly higher height (*p* < 0.001), weight (*p* < 0.001), BMI (*p* = 0.039), and bone age (*p* < 0.001), indicating accelerated growth and skeletal maturation compared to the control group. These findings indicate that CPP is associated with distinct physical development patterns that can help differentiate it from normal puberty.

**Table 1:** Clinical characteristics at baseline of the girls in the two groups.

Items	Control group (*n* = 116)	CPP group (*n* = 129)	p-Value
chronological age (years)	7.0 (7.0–8.0)	7.0 (7.0–8.0)	0.991
Height (cm)	123.00 (120.00–125.50)	130.70 (124.70–136.15)	<0.001
Weight (kg)	24.20 (20.90–26.10)	27.30 (23.90–31.80)	<0.001
BMI (kg/m^2^)	16.10 (14.47–17.05)	16.30 (15.44–17.65)	0.039
bone age (years)	7.64 (7.10–8.20)	9.64 (8.65–10.13)	<0.001

Values of *p* < 0.05 were considered statistically significant. BMI: body mass index.

### Elevated luteinizing hormone, kisspeptin, and estradiol characterize CPP

To further explore the hormonal differences underlying these physical changes, serum biomarker analysis was performed. The CPP group exhibited significantly higher levels of luteinizing hormone (2.51 mIU/mL vs. 0.23 mIU/mL, *p* < 0.001), kisspeptin (1.59 μg/L vs. 0.96 μg/L, *p* < 0.001), and estradiol (25.86 pg/mL vs. 13.41 pg/mL, *p* < 0.001) compared to the control group (Table 2). In contrast, vitamin D levels were significantly lower in the CPP group (20.13 ng/mL vs. 25.90 ng/mL, *p* < 0.001), suggesting an inverse relationship between vitamin D and markers of CPP. No significant differences were observed in progesterone and prolactin levels between the two groups. These biomarker patterns reflect distinct endocrine profiles in girls with CPP, with elevated gonadotropins and sex hormones as defining characteristics.

**Table 2:** Serum levels of luteinizing hormone, progesterone, prolactin, kisspeptin, vitamin D and estradiol in the two groups.

Items	Control group (*n* = 116)	CPP group (*n* = 129)	p-value
Luteinizing hormone (mIU/mL)	0.23 (0.08–0.45)	2.51 (0.69–3.84)	<0.001
Progesterone (ng/mL)	0.36 (0.21–0.73)	0.46 (0.26–0.87)	0.135
Prolactin (ng/mL)	9.75 (7.11–11.98)	8.89 (5.55–18.33)	0.814
Kisspeptin (μg/L)	0.96 (0.52–1.49)	1.59 (0.53–2.39)	<0.001
Vitamin D (ng/ml)	25.90 (22.64–31.17)	20.13 (16.94–24.11)	<0.001
Estradiol (pg/mL)	13.41 (9.86–16.98)	25.86 (18.45–33.18)	<0.001

Values of *p* < 0.05 were considered statistically significant.

### Diagnostic performance of biomarkers is enhanced by combining multiple indicators

**Figure 2:**
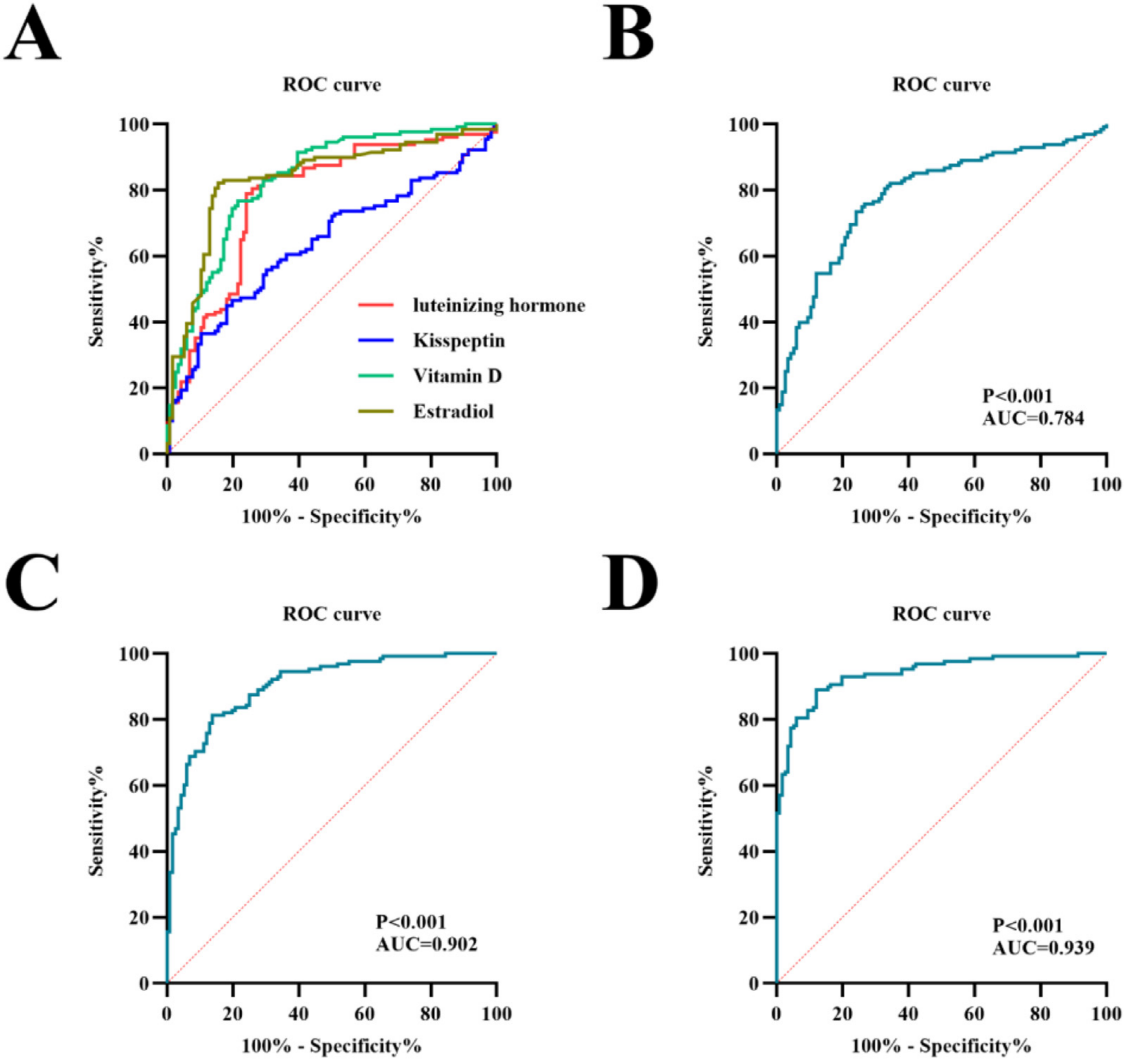
ROC curves for the control group and the CPP group and the combined auxiliary diagnostic models. (A) ROC curves for luteinizing hormone, kisspeptin, vitamin D and estradiol. (B) ROC curve for model 1. (C) ROC curve for model 2. (D) ROC curve for model 3. ROC, receiver operating characteristic.

Building on these biomarker differences, ROC curve analysis was conducted to evaluate the diagnostic utility of individual biomarkers and their combined models. Among the individual biomarkers, luteinizing hormone and estradiol demonstrated the highest diagnostic accuracy (Figure. 2A). Recognizing the potential for improved performance, three biomarker models were developed by combining various indicators. Model 1, which included luteinizing hormone and kisspeptin, achieved an AUC of 0.784 (95 % CI: 0.726–0.842) with a sensitivity of 73.44 % and specificity of 75.86 %. Adding vitamin D in Model 2 further improved diagnostic performance, achieving an AUC of 0.902 (95 % CI: 0.864–0.940), sensitivity of 81.25 %, and specificity of 86.21 % (Figure. 2B–2C). Model 3, which incorporated estradiol in addition to the other biomarkers, exhibited the highest diagnostic performance with an AUC of 0.939 (95 % CI: 0.910–0.968), sensitivity of 89.06 %, and specificity of 87.93 % (Figure. 2D). These results demonstrate that combining multiple biomarkers significantly enhances diagnostic precision for CPP.

### Combining biomarkers provides the best diagnostic utility for CPP

A comparison of the diagnostic models is presented in Table 3. While Model 1 provided moderate diagnostic accuracy, the inclusion of vitamin D in Model 2 resulted in a notable improvement in both sensitivity and specificity. Model 3, which added estradiol to the biomarker panel, outperformed the other models, offering the most accurate tool for CPP diagnosis. In addition, since the two indicators, progesterone and prolactin, showed no significant difference, they were not included in the final model. These findings highlight the value of integrating multiple biomarkers into diagnostic models, emphasizing their utility in clinical settings for improving the diagnosis of CPP. The results demonstrate that Model 3 represents a non-invasive, efficient, and highly accurate diagnostic approach, offering a promising alternative to traditional methods.

**Table 3:** Characteristics of the auxiliary diagnostic models.

Model	Included indicators	AUC	95 % CI	p-value	Sensitivity ( %)	Specificity ( %)	Cut-off value
Model 1	luteinizing hormone, kisspeptin	0.784	0.726–0.842	<0.001	73.44	75.86	0.493
Model 2	luteinizing hormone, kisspeptin, vitamin D	0.902	0.864–0.940	<0.001	81.25	86.21	0.675
Model 3	luteinizing hormone, kisspeptin, vitamin D, estradiol	0.939	0.910–0.968	<0.001	89.06	87.93	0.770

Values of *p* < 0.05 were considered statistically significant.

AUC, area under the curve; CI, confidence interval.

Since the two indicators, progesterone and prolactin, showed no significant difference, they were not included in the final model.

## Discussion

This study evaluated the diagnostic utility of key serum biomarkers and their combinations in distinguishing CPP from normal puberty. The present findings reveal significant differences in clinical and biochemical profiles between the CPP and control groups, highlighting the potential of integrating biomarkers into diagnostic models. These results address the limitations of traditional methods, such as the GnRH stimulation test, which, while effective, is invasive, time-consuming, and often impractical in routine clinical settings [4,5].

The clinical characteristics observed in this study, including higher height, weight, BMI, and bone age in the CPP group, align with previous research documenting accelerated growth and skeletal maturation in CPP patients [1,8]. However, while these characteristics support clinical suspicion of CPP, their specificity is limited due to overlap with other conditions such as premature thelarche or non-progressive puberty [6]. This underscores the importance of incorporating reliable biomarkers into diagnostic frameworks to improve accuracy and specificity.

Among the biomarkers analyzed, luteinizing hormone, kisspeptin, and estradiol were significantly elevated in girls with CPP, while vitamin D levels were notably reduced. These findings are consistent with earlier studies highlighting the role of these hormones in pubertal activation and progression [7,9]. Kisspeptin, a central regulator of pubertal onset, demonstrated particular diagnostic potential, consistent with previous studies showing its elevated levels in CPP cases [10]. Vitamin D, inversely associated with pubertal progression, provides additional diagnostic insight to this study, further validating earlier research suggesting its association with delayed puberty onset [11,15]. The inclusion of vitamin D in biomarker models represents an underexplored area that enhances the diagnostic framework presented in this study.

The ROC curve analysis revealed the limitations of individual biomarkers when used in isolation. Luteinizing hormone and estradiol demonstrated moderate diagnostic accuracy, consistent with earlier studies reporting AUC values of 0.7–0.8 for these markers [8,16]. However, combining biomarkers into models significantly improved diagnostic performance. Model 3, which included luteinizing hormone, kisspeptin, vitamin D, and estradiol, achieved the highest accuracy with an AUC of 0.939, surpassing most previously reported single-marker analyses. The diagnostic improvements achieved by integrating biomarkers align with emerging evidence suggesting that biomarker combinations provide greater sensitivity and specificity compared to single markers [13]. Although the current study has indicated a cut-off value for Model 3 of 0.770, caution should be taken when applying this threshold in guiding similar studies, given limitations such as the retrospective nature and single-center design of the current study. Currently, this model may serve as a screening tool for GnRH testing - individuals with high scores should undergo GnRH testing for CPP diagnosis, while those with low scores may be suitable for clinical observation. Future large-scale studies will be conducted to evaluate whether this model could replace the gold standard GnRH test.

Compared to previous research, this study provides a more comprehensive evaluation of integrated biomarker models. While earlier studies focused on traditional markers like luteinizing hormone and estradiol, this study incorporated kisspeptin and vitamin D, demonstrating their combined utility in enhancing diagnostic accuracy [10]. By addressing gaps in prior research, this study offers a novel and practical approach for diagnosing CPP.

Despite the promising results, certain limitations should be acknowledged. The retrospective nature of this study may introduce biases related to data collection and selection criteria [17]. In addition, the single-center design may limit the generalizability of these findings. Larger, multicenter studies are needed to validate these biomarker models across diverse populations. Furthermore, the cost-effectiveness and clinical feasibility of implementing these models in routine practice remain areas for future investigation [18].

## Conclusions

In conclusion, this study highlights the value of combining multiple biomarkers, including luteinizing hormone, kisspeptin, vitamin D, and estradiol, to improve the accuracy of CPP diagnosis. Model 3, which demonstrated superior sensitivity and specificity, offers a robust and non-invasive diagnostic tool for CPP. By incorporating a broader range of biomarkers and integrating them into clinically relevant models, this study addresses limitations in previous research and advances the field of biomarker-based diagnostics for CPP [19]. Future research should focus on validating these models in larger cohorts and assessing their implementation in clinical practice to optimize the diagnosis and management of CPP [20,21].

## Funding

None.

## Statement of ethics

The study was approved by Zibo Central Hospital; the approval number was 2010-SMY. The study was performed in strict accordance with the Declaration of Helsinki, Ethical Principles for Medical Research Involving Human Subjects.

## Informed consent

The need for informed consent was waived by Zibo Central Hospital.

## Data availability

The data are available from Xiaopeng Zhang upon reasonable request.

## Conflicts of interest

The authors declare no conflicts of interest.
